# “Functional trapezius muscle reconstruction after resection of a desmoid tumor using an innervated gracilis free flap”

**DOI:** 10.1080/23320885.2022.2062363

**Published:** 2022-08-15

**Authors:** Federico De Michele, Olindo Massarelli, Mara Franza, Vittorio Gebbia, Salvatore D’Arpa

**Affiliations:** ^a^ Department of Surgical, Oncological and Oral Sciences (Di.Chir.On.S.), Plastic and Reconstructive Surgery, Università degli Studi di Palermo, Palermo, Italy; ^b^ UOC di Chirurgia Maxillo Facciale. Azienda Ospedaliero Universitaria di Siena, Siena, Italy; ^c^ Dipartimento PROMISE, Università degli Studi di Palermo, Palermo, Italy; ^d^ Supervisor TDA Equipe, Private Practice, Palermo, Italy; ^e^ Program Director, Residency School in Plastic and Reconstructive Surgery, International University of Gorazde BH, Gorazde, Bosnia and Herzegovina

**Keywords:** Innervated gracilis free flap, trapezius functional reconstruction, desmoid tumor

## Abstract

Desmoid tumors are characterized by indolent growth, progressive invasion of surrounding tissues and a high rate of relapse. We present the case of a desmoid tumor rising from the trapezius of a young woman. Following resection, we performed a functional reconstruction using an innervated gracilis free flap.

## Introduction

A desmoid tumor (DT), also known as desmoid-type fibromatosis, is a rare benign neoplasm characterized by monoclonal growth of fibroblasts with the progressive invasion of soft tissues, a high rate of recurrence following treatment and the inability to metastasize. It can occur in any part of the body, with those arising in the limbs and trunk portending the poorest prognosis [1].

Active surveillance is now considered by most authors the best way to deal with asymptomatic disease [2–6]. On the other hand, surgery is often necessary for the management of symptomatic desmoid tumors and for those arising in structures that play an important role either for function or aesthetics.

## Case report

**Figure 1.**
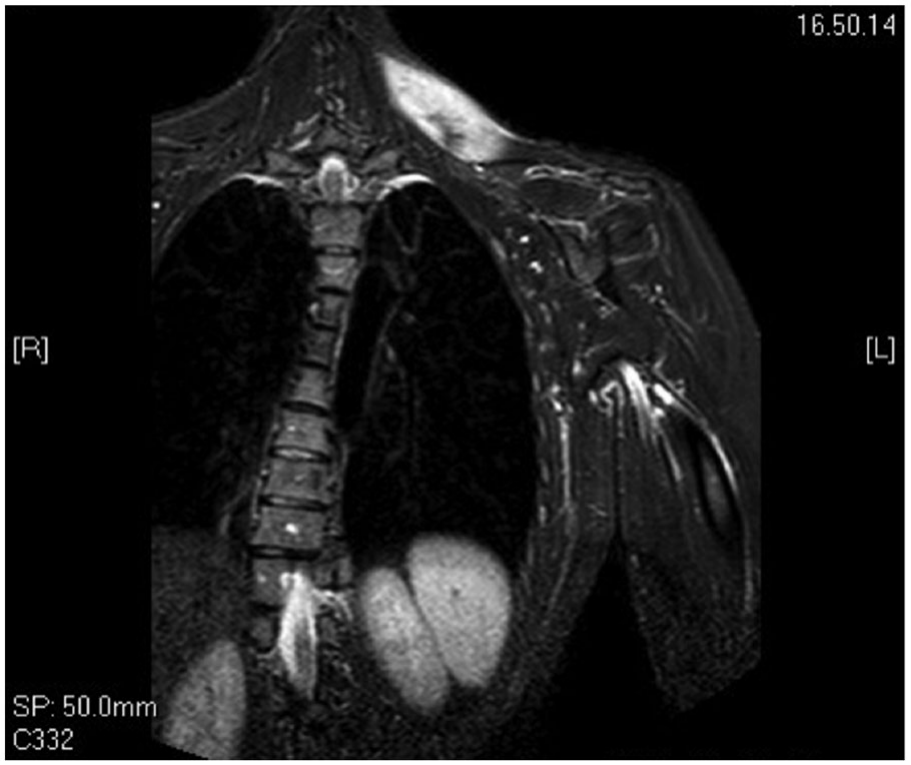
Pre-operative MRI shows the mass in the upper third of the trapezius.

A 34 years old woman was admitted to our institute for a soft palpable mass localized in her left trapezius. She complained about localized discomfort and pain that interfered with her daily activities. In order to better characterize the nature of the mass and its anatomical boundaries, an MRI (Figure 1) was performed which showed an intramuscular lesion of 8 cm × 4 cm infiltrating the cervical portion of the left trapezius without any involvement of the overlying subcutaneous tissues and skin. We subsequently performed an incisional biopsy that established the diagnosis of sporadic DT.

We discussed with the patient the possibility of a watchful follow-up before considering surgery. She opted for surgery in the first place, which we considered a reasonable choice in consideration of the symptoms she complained about.

Under general anesthesia, we performed an en-bloc resection of the mass with macroscopic margins of resection of about 1 cm. The mass was located in the upper third of the trapezius at the base of the neck, thus resection would involve the neuromuscular hilum leading to paralysis of the muscle, a condition seen after neck dissections with the sacrifice of the spinal accessory nerve [7]. Functional reconstruction was thus deemed necessary in order to preserve shoulder function and, secondarily, shape. We opted for an innervated musculocutaneous gracilis free flap because of its expendability, its similarity in shape with the resected specimen and its well-established role in head and neck muscle reanimations [8,9].

The upper third of the trapezius was resected en-bloc with the overlying skin including the scar from the previous biopsy. The spinal accessory nerve was prepared as close to the muscle hilum as possible and so were the transverse cervical vessels. The right transverse musculocutaneous gracilis free flap was transferred to the defect. The vascular anastomosis was performed end to end between the transverse cervical artery and vein and the gracilis pedicle. Neurorraphy was performed end to end between the spinal accessory nerve and the obturator nerve. The flap survived completely and the post-operative period was uneventful.

Three months following surgery the gracilis muscle recovered its contractility, as demonstrated by the follow-up EMG. After rehabilitation, the patient regained full range of motion and was able to go back to her daily routine, including swimming, her favourite hobby, without any restriction six months postoperatively. Pain and discomfort disappeared completely.

**Figure 2.**
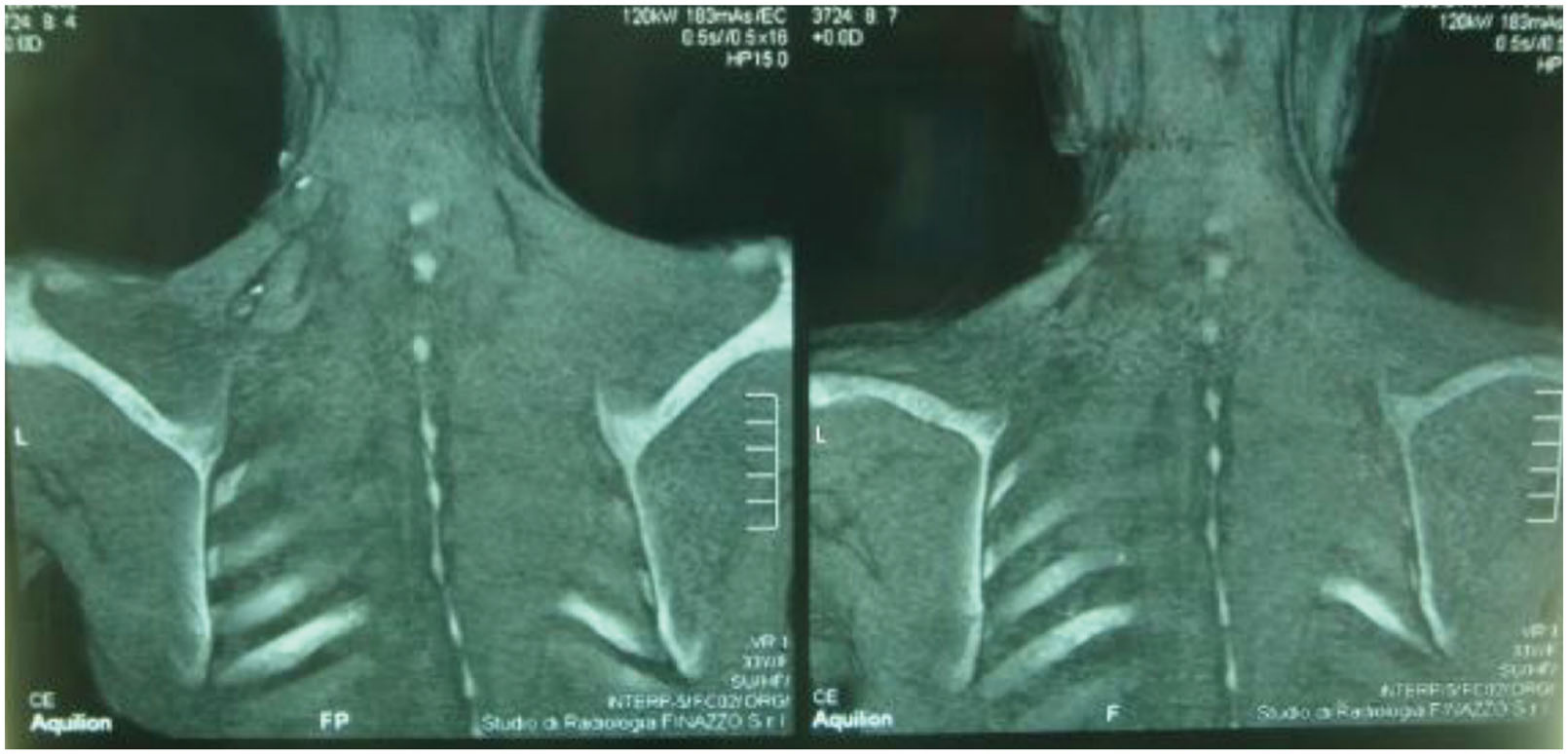
MRI control at 1 year post-op, showing perfect integration of the flap at the recipient site.

**Figure 3.**
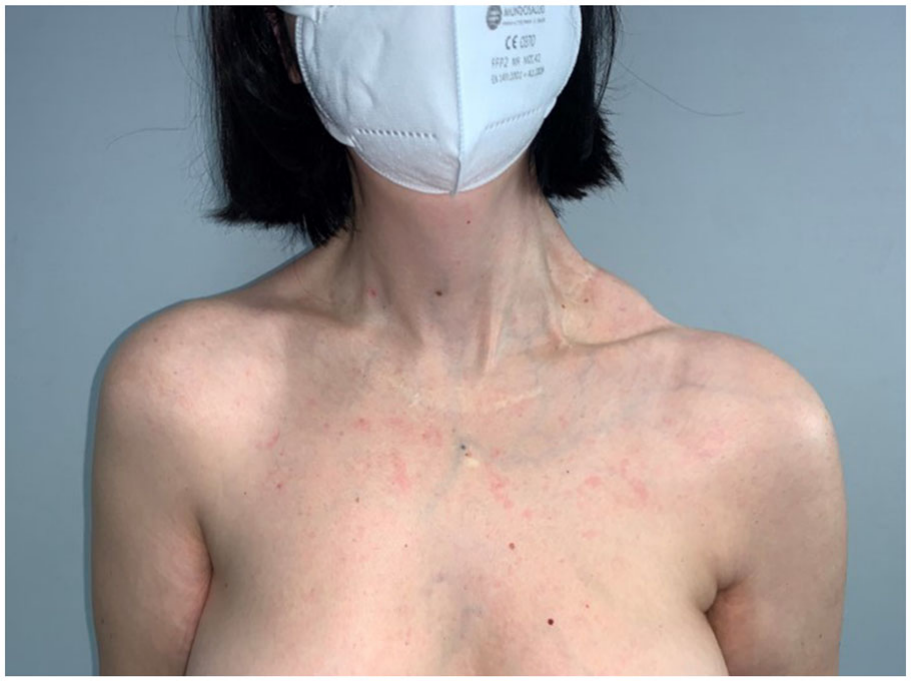
Clinical follow-up at 10 years.

**Figure 4.**
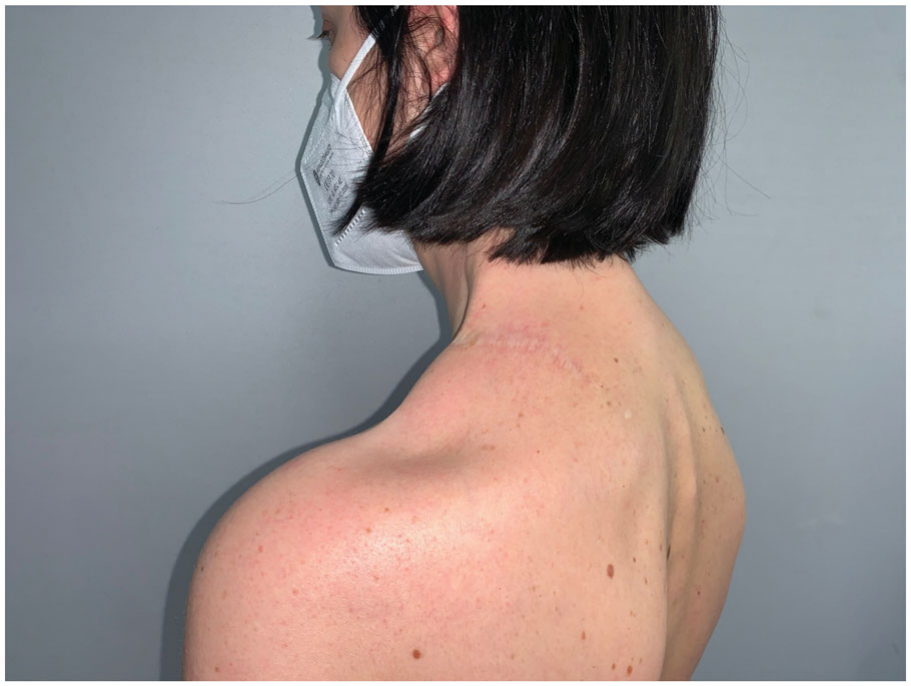
Clinical follow-up at 10 years.

**Figure 5.**
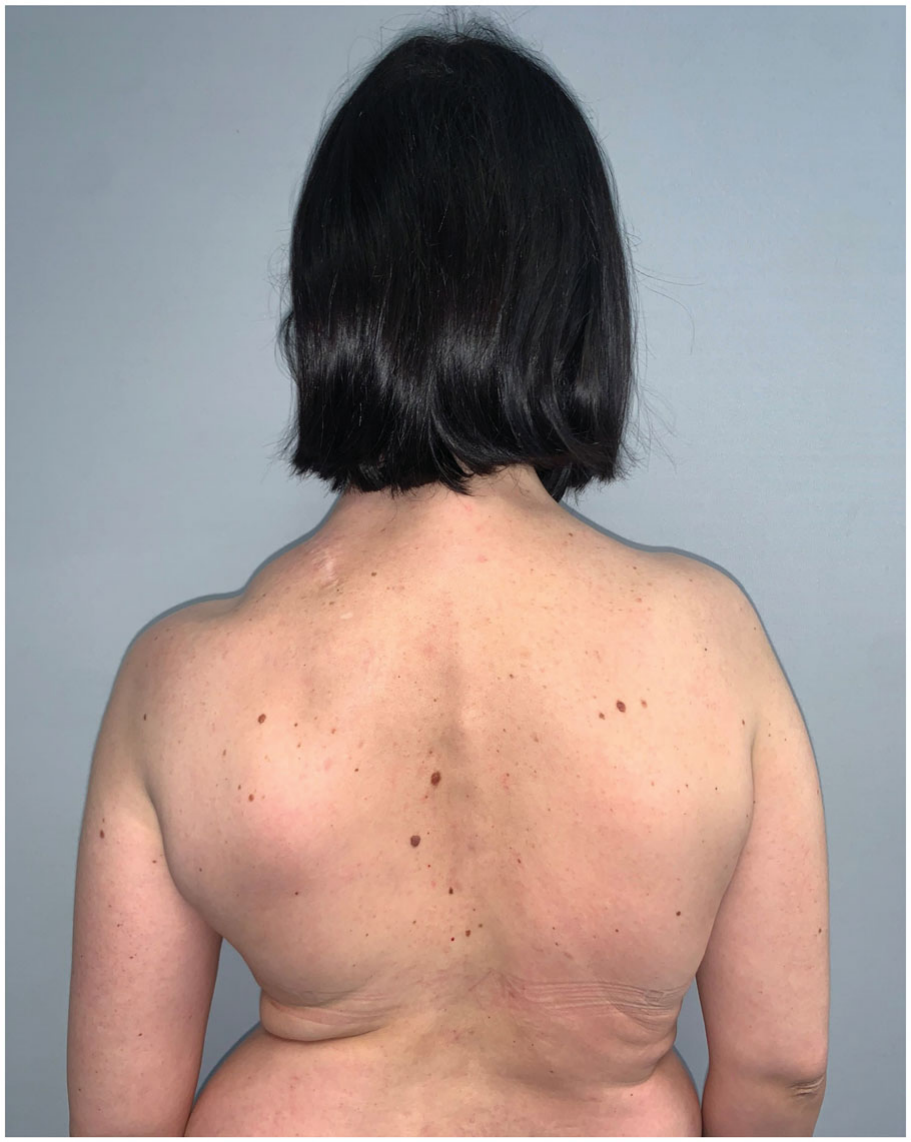
Clinical follow-up at 10 years.

**Figure 6.**
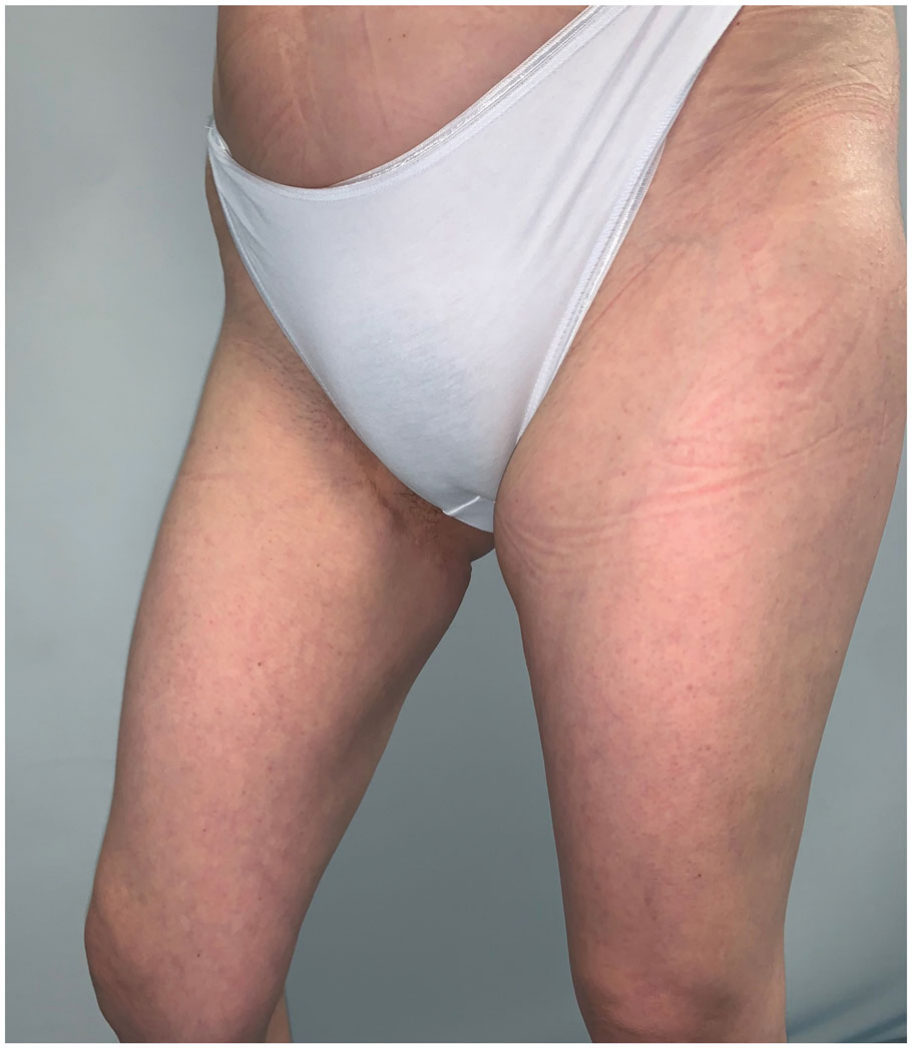
Clinical follow-up at 10 years.

After 9 months, to improve the cosmetic result and obtain a more attractive contour of the neck area, a skin expander was implanted in the subcutaneous region adjacent to the flap and progressively inflated until enough skin was gained in order to replace the flap island and reduce dimensions of the scar. Three months later the flap’s skin was resected and the resulting defect was covered with the expanded skin. The final aesthetic result was satisfying, consisting of just one linear eutrophic scar of about 7 cm. After a 7-year follow-up based on MRI and clinical evaluations, no signs of recurrence were observed (Figure 2). A 10 years of clinical follow-up shows a great aesthetic result both in recipient and donor site (Figure 3–6) and almost total functional recovery of the trapezius muscle (Supplementary Videos 1–3).

## Discussion

The desmoid tumor usually presents as an indolent, asymptomatic, growing mass. Diagnostic workup usually begins with imaging, with MRI being the mainstay. The suspicion of DT is then confirmed by core biopsy and is based on the detection of nuclear accumulation of b-catenin protein caused by activating mutations in the b-catenin gene [1].

Given the wide spectrum of clinical presentations and variable progression of the disease, treatment should be tailored to each patient; options encompass surgery, radiotherapy, medical therapy and isolated limb perfusion, the latter being reserved for DT of the extremities.

In recent series, up to 50% of asymptomatic patients had no progression of disease at 5 years and in 20–30% of the regression of disease was observed [3,10]. Hence, for asymptomatic patients, close surveillance has recently gained great popularity worldwide as the first-line approach. In particular, patients will undergo yearly follow-ups based on clinical assessment and contrast MRI. If progressive growth is confirmed at two or more consecutive follow-ups then treatment should be considered [1,6].

Selection of the best treatment option should be based on tumor location, dimensions, age and functional necessities of the patient. Surgery is still considered the first-line approach for intraabdominal DT [1,6] while in other sites medical therapy or radiotherapy alone can be considered in order to reduce treatment morbidity [3,6]. In particular, for DT of the extremities and girdle, the benefits of surgical resection should always be weighed against postoperative impairment. Local recurrence rates can be as high as 19–75%, but in most studies below 50% [11]. Currently, there is no consensus regarding the minimal surgical margin. Resections with positive margins or with free margins ≤ 1 mm are associated with a higher rate of recurrence [12,13]. On the other hand, once negative margins ≥ 1 mm are obtained, further widening of the resection in order to achieve larger disease-free margins doesn’t seem to consistently affect the probability of local recurrence. Yet whenever possible the surgeon should seek radical resections in order to reduce the chance of local recurrence [13]. However, some authors advocate that preserving functionality and minimizing surgical morbidity should always prevail over any attempt to obtain wide margins of resection [11]. Thus radical excisions involving bone or major neurovascular structures should be avoided [12]. Unfortunately in our case, the tumor happened to involve the cervical portion of the trapezius so surgical resection would have necessarily involved the trapezius hilum and therefore lead to muscle paralysis.

Since the morbidity of upper trapezius palsy following neck dissection with spinal accessory nerve sacrifice is well known, we sought a functional restoration of the upper trapezius by means of an innervated gracilis free flap [7,14]. Adequate muscle tensioning and precise technique enabled us to restore most of the trapezius strength minimizing postoperative morbidity. The outcome of surgical treatment was excellent, suggesting that when adequate reconstructive techniques to restore function are available, the threshold to consider surgical treatment of DT can be lowered. As described by Ihara et al., another optimal donor site suitable for reconstruction of the trapezius muscle can be the latissimus dorsi [15]. We believe that the gracilis is the best reconstructive option for small and medium-size defects mostly because of the minimal morbidity of the donor site and its use in reconstructions of the head and neck district is well established. In addition, it has the advantage that it can be comfortably harvested by a second surgical team while the first one takes care of the resection. The latissimus dorsi is a broad and flat trunk muscle that is closer in features to the trapezius and has been described as a pedicled flap for its reconstruction. Its shape and dimensions make it a valid option for the reconstructions of larger defects

## Conclusions

The innervated gracilis free flap represents a great choice for functional reconstruction of small and medium defects of trapezius muscle following tumor resection. The availability of muscular innervated tissue with minimal donor site morbidity, allows the surgeon to perform radical resection with greater disease-free margins and better control of disease without concern about reconstruction options. However, the rarity of the disease and the related prognosis requires more evidence to outline a defined therapeutic and reconstructive algorithm.

## Supplementary Material

Supplemental Material
